# Global DNA Methylation Patterns Can Play a Role in Defining Terroir in Grapevine (*Vitis vinifera* cv. Shiraz)

**DOI:** 10.3389/fpls.2017.01860

**Published:** 2017-10-30

**Authors:** Huahan Xie, Moumouni Konate, Na Sai, Kiflu G. Tesfamicael, Timothy Cavagnaro, Matthew Gilliham, James Breen, Andrew Metcalfe, John R. Stephen, Roberta De Bei, Cassandra Collins, Carlos M. R. Lopez

**Affiliations:** ^1^Environmental Epigenetics and Genetics Group, University of Adelaide, Adelaide, SA, Australia; ^2^The Waite Research Institute and The School of Agriculture, Food and Wine, University of Adelaide, Adelaide, SA, Australia; ^3^The ARC Centre of Excellence in Plant Energy Biology, University of Adelaide, Adelaide, SA, Australia; ^4^Robinson Research Institute, University of Adelaide, Adelaide, SA, Australia; ^5^Bioinformatics Hub, School of Biological Sciences, University of Adelaide, Adelaide, SA, Australia; ^6^School of Mathematical Sciences, University of Adelaide, Adelaide, SA, Australia; ^7^Plant Genomics Centre, Australian Genome Research Facility Ltd., Adelaide, SA, Australia

**Keywords:** environmental epigenetics, DNA methylation, terroir, MSAP, msGBS, *Vitis vinifera*, Shiraz, Barossa

## Abstract

Understanding how grapevines perceive and adapt to different environments will provide us with an insight into how to better manage crop quality. Mounting evidence suggests that epigenetic mechanisms are a key interface between the environment and the genotype that ultimately affect the plant’s phenotype. Moreover, it is now widely accepted that epigenetic mechanisms are a source of useful variability during crop varietal selection that could affect crop performance. While the contribution of DNA methylation to plant performance has been extensively studied in other major crops, very little work has been done in grapevine. To study the genetic and epigenetic diversity across 22 vineyards planted with the cultivar Shiraz in six wine sub-regions of the Barossa, South Australia. Methylation sensitive amplified polymorphisms (MSAPs) were used to obtain global patterns of DNA methylation. The observed epigenetic profiles showed a high level of differentiation that grouped vineyards by their area of provenance despite the low genetic differentiation between vineyards and sub-regions. Pairwise epigenetic distances between vineyards indicate that the main contributor (23–24%) to the detected variability is associated to the distribution of the vineyards on the N–S axis. Analysis of the methylation profiles of vineyards pruned with the same system increased the positive correlation observed between geographic distance and epigenetic distance suggesting that pruning system affects inter-vineyard epigenetic differentiation. Finally, methylation sensitive genotyping by sequencing identified 3,598 differentially methylated genes in grapevine leaves that were assigned to 1,144 unique gene ontology terms of which 8.6% were associated with response to environmental stimulus. Our results suggest that DNA methylation differences between vineyards and sub-regions within The Barossa are influenced both by the geographic location and, to a lesser extent, by pruning system. Finally, we discuss how epigenetic variability can be used as a tool to understand and potentially modulate terroir in grapevine.

## Introduction

The ability of plants to produce alternative phenotypes in response to changing environments is known as phenotypic plasticity (Pigliucci, 2005). Genotypes with this characteristic are able to produce a variety of phenotypes including improved growth and reproduction (Lacaze et al., 2009). Grapevine (*Vitis vinifera* L.) is a highly plastic crop that exhibits large differences in fruit composition from a given variety depending upon the environmental conditions of the vineyard of origin (Dal Santo et al., 2016). Fruit traits that affect wine quality are thought to be largely driven by the interaction of a grapevine’s genetic characteristics with environmental factors (i.e., climate, soil, and topography) and vineyard management (Robinson et al., 2012). The grapevine cycle extends for two seasons, with buds formed in a specific year giving rise to shoots that will carry fruit the next year (Keller et al., 2010). Environmental cues over two seasons can impact on yield (fruit quantity) and fruit composition by influencing the formation of the inflorescence primordia (Buttrose, 1974), flowering and fruitset (Petrie and Clingeleffer, 2005). Temperature and water availability are also known to influence sugar concentration, acidity, pH, color, and other characteristics in the fruit (Adams, 2006; Downey et al., 2006). Moreover, climate change predictions of elevated CO_2_ and rising temperature are also likely to have an effect on the grapevine reproductive cycle and on fruit composition (Parra et al., 2010). All these variables, in conjunction with the wine making process, give a wine its distinctive character. The impact of the environment on grape composition and subsequent wine excellence has given rise to the concept of ‘terroir,’ a French term referring to *terre*, “land” (Fanet and Brutton, 2004).

Terroir is defined as the interaction between the physical and biological environment and applied viticultural and oenological practices that lead to unique characteristics in a final wine (Seguin, 1986). Extensive studies have been published on terroir, but generally, these focus on a single parameter such as climatic factors, soil structure, or soil microbiology (Harrison, 2000; Tonietto and Carbonneau, 2004). However, studying only one environmental parameter does not provide an entire understanding of how wine quality is influenced by terroir (van Leeuwen et al., 2004). A significant amount of work has also been published on the genetic basis of fruit composition in grapevines (e.g., Doligez et al., 2002). Despite these insights, further research is required on the molecular changes that are involved in the vine interaction with its environment.

One of the molecular changes worth investigating relates to environmentally induced epigenetic modifications. In fact, phenotypic plasticity has been previously associated to epigenetic variation (Vogt, 2015). Interestingly, analysis of epigenetic diversity has been shown to be more effective in discriminating inter-clonal variability in grapevine than the use of purely genetic molecular markers such as simple sequence repeats (SSRs) or amplified fragment length polymorphisms (AFLPs) (Imazio et al., 2002; Schellenbaum et al., 2008; Ocaña et al., 2013). Epigenetic mechanisms refer to potentially heritable (via mitosis or meiosis) molecular changes that affect gene expression leading to differences in phenotype without changing the organism DNA sequence (Jaenisch and Bird, 2003; Haig, 2004). Such mechanisms are involved in the control of a range of plant processes, including developmental control (Daccord et al., 2017), genomic imprinting (Köhler et al., 2012), and response to stress (Yaish et al., 2011; Tricker et al., 2012). It is now also widely accepted that epigenetic mechanisms have been the source of useful variability during crop varietal selection (Amoah et al., 2012; Bloomfield et al., 2014; Rodríguez López and Wilkinson, 2015).

Multiple environmental cues have been shown to induce persistent changes in epigenetic modifications, resulting in an epigenetic priming that can act over multiple vegetative (Kumar et al., 2016) or sexual generations (Tricker et al., 2012). Although whether environmentally induced epialleles have any effect on the phenotypes of future generations remains a matter of debate, such priming is considered by some as an adaptive strategy by which plants use their memory of the environment to modify their phenotypes to adapt to subsequent conditions (Kelly et al., 2012; Tricker et al., 2013a,b; Vogt, 2015). It is commonly accepted that DNA methylation constitutes an adaptation strategy to the environment (YunLei et al., 2009), and that changes in DNA methylation can produce altered phenotypes (Zhang et al., 2006; Herrera and Bazaga, 2011; Iqbal et al., 2011). Moreover, epigenetic mechanisms are now considered as potential drivers of rapid adaptation to the environmental variability (Bräutigam et al., 2013). These processes facilitate adaptation by regulating the expression of genes controlling phenotypic plasticity (Richards, 2006; Bossdorf et al., 2008) early in adaptive walks (Kronholm and Collins, 2016) but also by releasing cryptic genetic variation and/or facilitating mutations in functional loci over longer-term timescales (O’Dea et al., 2016). To this extent, there have been extensive studies establishing a link between DNA methylation in plants and environmental conditions both in wild (Fonseca Lira-Medeiros et al., 2010; Herrera and Bazaga, 2010; Alonso et al., 2016) and cultivated species (Zheng et al., 2017).

All major epigenetic mechanisms, DNA methylation, histone modifications, and RNA interference, are present in plants (Pikaard and Mittelsten Scheid, 2014; Holoch and Moazed, 2015; Wendte and Pikaard, 2017). In plants, DNA methylation (5mC) occurs at different cytosine contexts (CpG, CpHpG, or CpHpH) (H = A, T or C) (Richards, 1997; Baulcombe and Dean, 2014; Niederhuth and Schmitz, 2017) and it is induced, maintained or removed by different classes of methyltransferase in conjunction with environmental and developmental cues (Law and Jacobsen, 2010; Baulcombe and Dean, 2014). The contribution of DNA methylation to plant performance has been extensively studied in model organisms and some annual crops (Rodríguez López and Wilkinson, 2015). However, we are only beginning to understand how long-living plants, such as grapevines, use epigenetic mechanisms to adapt to changing environments (Fortes and Gallusci, 2017). Effects of environmental conditions on non-annual crops performance can be very difficult to evaluate since many environmental factors interact over the life of the plant to ultimately contribute toward the plant’s phenotype (Fortes and Gallusci, 2017). Although epigenetic mechanisms have been shown to act as a memory of the organism’s growing environment during mitotic division (Morao et al., 2016), even after vegetative propagation (Raj et al., 2011; Guarino et al., 2015), very few studies have focussed on DNA methylation changes in grapevine. The few known studies in this field used MSAPs (Reyna-López et al., 1997) for the detection of *in vitro* culture induced epigenetic somaclonal variability (Baránek et al., 2015), and the identification of commercial clones (Imazio et al., 2002; Schellenbaum et al., 2008; Ocaña et al., 2013).

In this study, we hypothesize that DNA methylation can play a role in defining terroir. To test this hypothesis, we investigated the effect of environmental and management conditions on DNA methylation variation in grapevine cultivar Shiraz across 22 vineyards representative of The Barossa wine zone (Australia) (Robinson and Sandercock, 2014) using MSAPs. Finally, we used msGBS to characterize the genomic context of the observed regional genetic and epigenetic variability.

## Materials and Methods

### Vineyard Selection and Plant Material

Vines from 22 commercial vineyards located in the iconic Barossa wine zone (The Barossa hereafter) (Australia) were included in this study. Vineyards were chosen based on the knowledge that they produce premium Shiraz wines that are representative of the climate, soil and management practices that are used in the Barossa sub-regions as described by the Barossa Grounds Project (Robinson and Sandercock, 2014) [i.e., Eden Valley (three vineyards) and Barossa Valley (19 vineyards) which included vineyards in the five distinctive sub-regions within the Barossa Valley Region: Northern Grounds (four vineyards), Central Grounds (four vineyards), Eastern Edge (four vineyards), Western Ridge (four vineyards), Southern Grounds (three vineyards)] (Supplementary Table S1). To simplify the nomenclature, the Eden Valley region, Northern, Central, Southern Grounds, Eastern Edge, and Western Ridge will be defined as sub-regions hereafter. All vineyards were planted with own-rooted vines of the cv. Shiraz. Ten vineyards were planted with clone SA 1654 (Whiting, 2003), one with clone BVRC30 (Whiting, 2003), one with clone PT15 Griffith (Farquhar, 2005) and 10 of ‘unknown’ clonal status (Supplementary Table S1).

Nine vines from three rows from each vineyard were selected and vines adjacent to missing vines, end of row vines and border rows were excluded, to prevent differences in competition effects between plants. Also, rows containing sampled plants were selected from each vineyard after discussion with vineyard managers to capture the variability in each vineyard. A total of 198 plants (nine plants per vineyard) were selected to capture the diversity from each vineyard. Leaf samples (first fully expanded leaf at bud burst, E-L 7) (Coombe, 1995) were collected from three nodes per plant and pooled into a single sample per plant. All samples were taken before dawn (between 10:00 pm and sunrise) to minimize variability associated with differences in plant water status (Williams and Araujo, 2002). Samples were immediately snap-frozen in liquid nitrogen in the vineyard and stored at -80°C until DNA extraction.

### DNA Isolation

Genomic DNA extractions from all 198 samples were performed using the three pooled leaves per plant powdered using an automatic mill grinder (Genogrinder). The obtained frozen powder was used for DNA extraction using the Oktopure automated DNA extraction platform (LGC Genomics GmbH) according to the manufacturer’s instructions. Isolated DNA was quantified using the Nanodrop 2000 spectrophotometer (Thermo Fisher Scientific, Wilmington, DE, United States). DNA final concentrations were normalized to 20 ng/μl using nanopure water (Eppendorf, Germany).

### Analysis of Genetic/Epigenetic Variability Using MSAP

Methylation sensitive amplified polymorphism analysis was performed as described by Rodríguez López et al. (2012). In brief, gDNA from 88 plants (four plants per vineyard) was digested with a combination of the restriction enzymes *Eco*RI and one of two DNA methylation sensitive isoschizomers (*Hpa*II or *Msp*I). Double stranded DNA adapters (See Supplementary Table S2 for the sequence of all oligonucleotides used) containing co-adhesive ends complementary to those generated by EcoRI and *Hpa*II/*Msp*I were ligated to the digested gDNA and then used as a template for the first of two consecutive selective PCR amplifications in which the primers were complementary to the adaptors but possessed unique 3′ overhangs. The second selective PCR amplification used primers containing 3′ overhangs previously tested on grapevine (Baránek et al., 2015). *Hpa*II/*Msp*I selective primer was 5′ end-labeled using a 6-FAM reporter molecule for fragment detection using capillary electrophoresis on a ABI PRISM 3130 (Applied Biosystems, Foster City, CA, United States) housed at the Australian Genome Research Facility Ltd., Adelaide, SA, Australia.

Generated electropherograms were visualized using GeneMapper Software v4 (Applied Biosystems, Foster City, CA, United States). A binary matrix containing presence (1) absence (0) epilocus information was generated for each enzyme combination (i.e., *Eco*RI/*Hpa*II and *Eco*RI/*Msp*I). MSAP fragment selection was limited to allelic sizes between 95 and 500 bp to reduce the potential impact of size co-migration during capillary electrophoresis (Caballero et al., 2008). Different levels of hierarchy were used to group the samples. Samples were first grouped according to vineyard of origin. Then, samples were divided into their sub-regions of origin. Finally, samples were further separated into groups according to clones and the vineyard management systems (i.e., pruning system used in their vineyard of origin) (Supplementary Table S1).

*Hpa*II and *Msp*I binary matrices were then used to compute Shannon’s Diversity Index implemented using *msap* R package (v. 1.1.8) (Pérez-Figueroa, 2013) and PCoA was estimated in all regions to determine and visualize the contribution to the observed molecular variability within regions of NML and of MSL (genetic and epigenetic variability, respectively) (Smouse et al., 2015).

GenAlex v 6.5 software (Peakall and Smouse, 2012) was used for PCoA in order to visualize the molecular differentiation between Barossa sub-regions detected using MSAP profiles generated after the restriction of gDNA with *Hpa*II or *Msp*I. We then used AMOVA to determine the structure of the observed variability using PCoA. Molecular differences between vineyards and regions was inferred as pairwise PhiPT distances (Michalakis and Excoffier, 1996).

Mantel test analysis (Hutchison and Templeton, 1999) was used to estimate the correlation between the calculated pairwise molecular distances with (1) the GeoD [i.e., Log(1 + GeoD (km)] and (2) differences in environmental variables among vineyards (i.e., vineyard altitude, regional average annual rainfall, regional growing season rainfall, regional mean January temperature, regional growing season temperature, and growing degree days). Mantel test was implemented in Genalex v 6.5 as described by Róis et al. (2013) and significance was assigned by random permutations tests (based on 9,999 replicates).

### Characterization of Genetic/Epigenetic Variability Using msGBS

Methylation sensitive genotyping by sequencing was performed as described by Kitimu et al. (2015). In brief, 200 ng of gDNA from nine samples from Northern, Central, and Southern Grounds (vineyards 1–4, 5–8, and 13–15, respectively) were digested using 8 U of HF-*Eco*RI and 8 U of *Msp*I (New England BioLabs Inc., Ipswich, MA, United States) in a volume of 20 μl containing 2 μl of NEB Smartcut buffer at 37°C for 2 h followed by enzyme inactivation at 65°C for 10 min. Sequencing adapters were ligated by adding 0.1 pmol of the *Msp*I adapters (uniquely barcoded for each of the 198 samples) and 15 pmol of the common *Eco*RI Y adapter (See Supplementary Table S2 for the sequence of all oligonucleotides used), 200 U of T4 ligase and T4 ligase buffer (New England BioLabs Inc., Ipswich, MA, United States) in a total volume of 40 μl at 24°C for 2 h followed by an enzyme inactivation step at 65°C for 10 min. Excess adapters were removed from ligation products using Agencourt AMPure XP beads (Beckman Coulter, Australia) at the ratio of 0.85 and following manufacturer’s instructions. Single sample msGBS libraries were then quantified using Qbit 3 (Thermo Fisher). A single library was generated by pooling 25 ng of DNA from each sample. Library was then amplified in eight separate PCR reactions (25 μl each) containing 10 μl of library DNA, 5 μl of 5x Q5 high fidelity buffer, 0.25 μl polymerase Q5 high fidelity, 1 μl of each forward and reverse common primers at 10 μM, 0.5 μl of 10 μM dNTP and 7.25 μl of pure sterile water. PCR amplification was performed in a Bio-Rad T100 thermocycler consisting of DNA denaturation at 98°C (30 s) and 10 cycles of 98°C (30 s), 62°C (20 s), and 72°C (30 s), followed by 72°C for 5 min. PCR products were then re-pooled and DNA fragments ranging between 200 and 350 bp in size were captured using the AMPure XP beads following manufacturer’s instructions. Libraries were sequenced using an Illumina NextSeq High Output 75 bp pair-end run (Illumina Inc., San Diego, CA, United States) at the Australian Genome Research Facility (AGRF, Adelaide, SA, Australia).

### msGBS Data Analysis

Analysis of genetic diversity between regions was performed by SNP calling using TASSEL 3 (Bradbury et al., 2007) on msGBS sequencing results. Only SNPs present in at least 80% of the samples were considered for analysis. PCA was implemented on TASSEL 3 using the selected SNPs. To identify any possible geographical genetic structure, the optimal number of genetic clusters present in the three regions were computed using BIC as effected by DAPC using adegenet 2.0.0^1^.

Identification of significant DMMs between regions was then computed using the package *msgbsR*^2^ (accessed on 26/08/2016). In brief, raw sequencing data was first demultiplexed using GBSX (Herten et al., 2015) and filtered to remove any reads that did not match the barcode sequence used for library construction. Following demultiplexing, paired-end reads were merged using bbmerge in bbtools package (Bushnell, 2016). Merged reads were next aligned to the 12X grapevine reference genome^3^. Alignment BAM files where then used to generate a read count matrix with marker sequence tags, and used as source data to perform subsequent analyses using *msgbsR* in the R environment (R Core Team, 2015). Finally, the presence of differential methylation between regions was inferred from the difference in the number of read counts from all sequenced *Msp*I containing loci that had at least 1 CPM reads and present in at least 15 samples per region. Significance threshold was set at Bonferroni adjusted *P*-value (or FDR) < 0.01 for difference in read CPM. The *log*FC (logarithm 2 of fold change) was computed to evaluate the intensity and direction of the region specific DNA methylation polymorphism.

To determine how the observed changes in DNA methylation between sub-regions were associated to protein coding genes, the distribution of DMMs was assessed around such genomic features, as defined in Ensembl database^4^, by tallying the number of DMMs between the TSS and the TES and within five 1 kb windows before the TSS and after TES of all *V. Vinifera* genes, using *bedtools* (Quinlan and Hall, 2010).

Genes within 5 kb of a DMM were referred to as DMGs. DMGs in each pairwise regional comparison were grouped into those showing hypermethylation or hypomethylation, and were next used separately for GO terms enrichment, using the R packages: *GO.db* (Carlson, 2016) and *annotate* (Gentleman, 2016). Significant GO terms were selected based on Bonferroni adjusted *P*-values at significance threshold of 0.05. Finally, GO terms containing DMGs in all three pairwise comparisons were visualized using REViGO (Supek et al., 2011).

## Results

**FIGURE 1:**
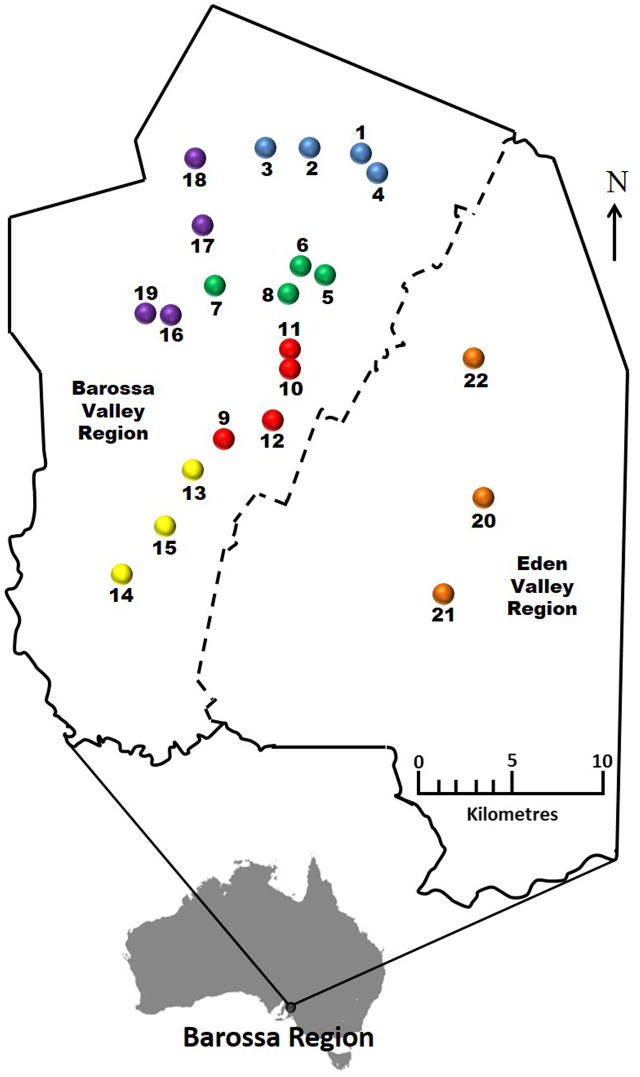
Selected Barossa region vineyard sites. Northern Grounds: Blue, Southern Grounds: Yellow, Central Grounds: Green, Eastern Edge: Red, Western Ridge: Purple, Eden Valley: Orange. Arrow indicates geographic north.

Analysis of MSAP profiles obtained from DNA extractions of the first fully expanded leaf of 88 individual vines selected from 22 commercial vineyards within the six Barossa sub-regions (**Figure 1** and Supplementary Table S1) yielded 215 fragments comprising 189 from *Msp*I and 211 from *Hpa*II, of which 80 and 84%, respectively, were polymorphic (i.e., not present in all the analyzed samples/replicates when restricted with one of the isoschizomers). Comparison of the *Hpa*II and *Msp*I banding patterns showed that in average, 42.1% of analyzed bands represented fully methylated or SNP containing loci, 22.3% represented hemimethylated loci, 19.6% represented un-methylated loci, and 18.1% represented loci containing internal cytosine methylation (Supplementary Table S3).

### Analysis of Genome/Methylome Differences within Wine Sub-regions in the Barossa

Principal coordinate analysis of the MSAP profiles generated from NML (genetic variability) and by MSL (epigenetic variability) (Pérez-Figueroa, 2013) revealed a higher separation between vineyards when using epigenetic information than when using genetic (**Supplementary Figure S1**). The capacity of both types of variability to differentiate between vineyards was more evident on vineyards in the Southern Grounds (**Supplementary Figures S2G,H**). Both PCoA analysis and Shannon’s diversity index showed significantly higher epigenetic than genetic diversity for all sub-regions (**Supplementary Figure S2** and **Table 1**). Among sub-regions, Southern Grounds had the highest epigenetic diversity (0.581 ± 0.124) and Western Ridge the lowest (0.536 ± 0.143). Genetic diversity showed the highest value in the Southern Grounds (0.374 ± 0.143) and the lowest in the Northern Grounds (0.240 ± 0.030).

**Table 1:** Analysis of genetic (NML) and epigenetic (MSL) diversity within the six Barossa Valley wine growing regions: Columns #MSL and #NML indicate the number of methylation sensitive loci, the number of non-methylated loci detected in plants analyzed in each region.

Region	#Vineyards	#Plants	#MSL	#NML	%Polym MSL	%Polym NML	Shannon Index	
							
							MSL	NML
**NG**	4	16	161	54	54	41	0.542 (0.119)	0.240 (0.030)
**CG**	4	16	169	46	50	37	0.552 (0.124)	0.242 (0.035)
**ER**	4	16	177	38	58	37	0.547 (0.138)	0.244 (0.038)
**SG**	3	12	150	65	57	34	0.581 (0.124)	0.374 (0.143)
**WE**	4	16	163	52	64	17	0.536 (0.133)	0.250 (0.048)
**EV**	3	16	158	57	63	33	0.573 (0.095)	0.287 (0.000)


*Columns %Polym MSL and %Polym NML show the percentage of polymorphic loci of each class per region. Shannon diversity indices are reported as mean (± SD). Wilcoxon rank test provides statistical support for all Shannon diversity indices (*P* < 0.0001).*

### Analysis of Genome/Methylome Differences between Wine Sub-regions in the Barossa

We used AMOVA (**Table 2**) to obtain an overview of the molecular variability between all the studied sub-regions. Overall, MSAP profiles generated using restriction enzyme *Msp*I achieved better separation between sub-regions than those generated using *Hpa*II. Of all 30 calculated molecular pairwise distances between sub-regions (PhiPTs), 25 were significant (*P* < 0.05) (**Table 2**). Calculated PhiPT values ranged from 0.115 (PhiPT of Northern Grounds vs Southern Grounds calculated using *Msp*I) and 0.012 (PhiPT of Central Grounds vs Eastern Edge calculated using *Hpa*II).

**Table 2:** Molecular distances (PhiPT) between Barossa Valley wine producing sub-regions.

	North	South	Central	East	West	Eden
North	_	0.115 (1e-04)	0.043 (8e-04)	0.062 (2e-04)	0.082 (1e-04)	0.069 (0.001)
South	0.028 (0.0059)	_	0.064 (1e-04)	0.042 (0.001)	0.027 (0.024)	**0.024 (0.073)**
Central	**0.012 (0.1085)**	0.025 (0.0079)	_	0.043 (1e-04)	0.060 (1e-04)	0.067 (2e-04)
East	0.025 (0.0043)	**0.012 (0.0712)**	0.015 (0.0474)	_	0.029 (0.004)	0.038 (0.0011)
West	0.039 (2e-04)	0.018 (0.0426)	0.033 (0.0001)	**0.013 (0.0651)**	_	0.024 (0.024)
Eden	0.056 (0.0001)	0.043 (4e-04)	**0.015 (0.0601)**	0.031 (0.0023)	0.031 (0.0016)	_


*PhiPT values were calculated using MSAP profiles generated from 88 grapevine plants (four individual plants per vineyard) using restriction enzyme combinations *Msp*I/*EcoR*I (above diagonal) and *Hpa*II/*EcoR*I (below diagonal). *P*-values (shown in parenthesis) were calculated based on 9,999 permutations. Pairwise regional comparisons showing not significantly different PhiPT values are highlighted in bold. A total of 22 vineyards were included in the analysis: Northern Grounds (4), Central Grounds (4), Southern Grounds (3), four vineyards in Eastern Edge (4), four vineyards in Western Ridge (4) and Eden valley (3).*

Analysis of molecular variance on MSAP profiles indicates that the majority of the observed variability is explained by differences within vineyards (81% using profiles generated with *Msp*I and 91% with *Hpa*II). A significant proportion of the total variability detected was associated to differences between vineyards (17% with *Msp*I and 8% with *Hpa*II) and 2 and 1% was due to differences between sub-regions (*Msp*I and *Hpa*II, respectively).

### Effect of Vineyard Location on Methylome Differentiation

**FIGURE 2:**
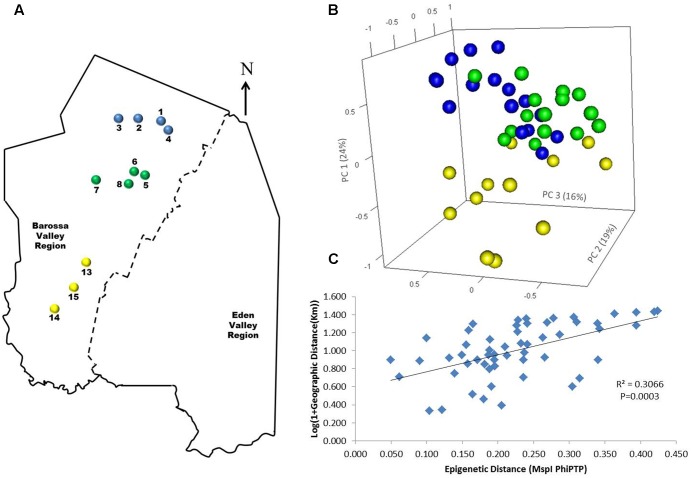
Analysis of the correlation between molecular differentiation and geographic distance (Km) of vineyards planted along the Barossa Valley North–South axis. **(A)** Location of the Barossa Valley vineyards from the three sub-regions distributed along the Barossa Valley North–South axis; Northern Grounds (blue), Central Grounds (green), and Southern Grounds (yellow). Arrow indicates the direction of geographic North. **(B)** PCoA representing genetic and epigenetic differences between leaf samples collected from four plants/vineyard. Percentage of the variability capture by each principal coordinate (PC) is shown in parenthesis. **(C)** Correlation between pairwise genetic/epigenetic distance (*Msp*I PhiPT) and geographical distance [Log(1 + GeoD) km] between vineyards. Shown equations are the correlation’s *R*^2^ and the Mantel test significance (*P*-value was estimated over 9,999 random permutations tests). PCoA and PhiPT for Mantel test were based on presence/absence of 215 loci obtained from MSAP profiles generated using *Msp*I.

**FIGURE 3:**
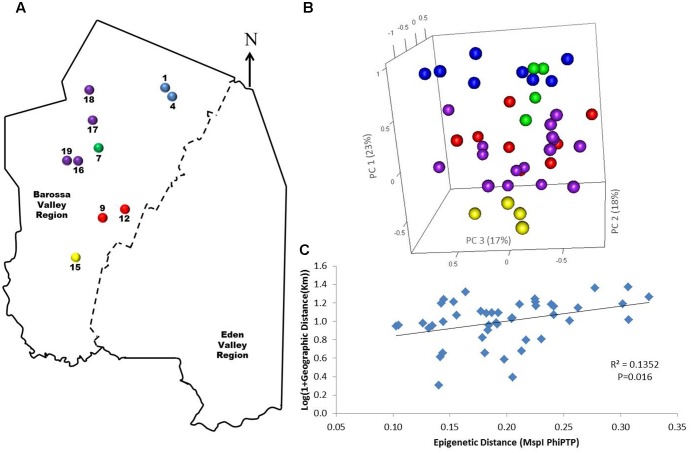
Analysis of the correlation between molecular differentiation and geographic distance (Km) of vineyards planted with clone 1654 in the Barossa region. **(A)** Location of the selected Barossa Valley vineyards from the three sub-regions distributed along the Barossa Valley North–South axis Northern Grounds (blue), Central Grounds (green), Eastern Edge (red), Southern Grounds (yellow), and Western Ridge (purple). Arrow indicates the direction of geographic North. **(B)** PCoA representing genetic/epigenetic differences between leaf samples collected from four plants/vineyard. Percentage of the variability captured by each PC is shown in parenthesis. **(C)** Correlation between pairwise genetic/epigenetic distance (*Msp*I PhiPT) and geographical distance [Log(1 + GeoD) (km)] between vineyards. Shown equations are the correlation’s *R*^2^ and the Mantel test significance (*P*-value was estimated over 9,999 random permutations tests). PCoA and PhiPT for Mantel test were based on presence/absence of 215 loci obtained from MSAP profiles generated using *Msp*I.

**FIGURE 4:**
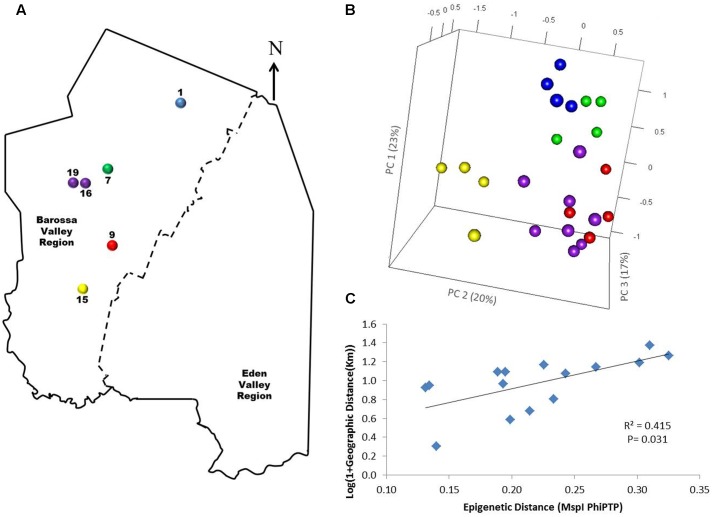
Analysis of the correlation between molecular differentiation and geographic distance (Km) of vineyards planted with clone 1654 in the Barossa Region and trained using the spur pruned method. **(A)** Location of the selected Barossa Valley vineyards: Northern Grounds (blue), Central Grounds (green), Eastern Edge (red), Southern Grounds (yellow), and Western Ridge (purple). Arrow indicates the direction of geographic North. **(B)** PCoA representing genetic/epigenetic differences between leaf samples collected from four plants/vineyard. Percentage of the variability capture by each PC is shown in parenthesis. **(C)** Correlation between pairwise genetic/epigenetic distance (*Msp*I PhiPT) and geographical distance [Log(1 + GeoD) (km)] between vineyards. Shown equations are the correlation’s *R*^2^ and the Mantel test significance (*P*-value was estimated over 9,999 random permutations tests). PCoA and PhiPT for Mantel test are based on presence/absence of 215 loci obtained from MSAP profiles generated using *Msp*I.

To determine if environmental differences between vineyards influenced the observed epigenetic differences we studied the vineyards’ pairwise geographic and molecular distances correlation. Vineyards located on the North–South axis of the Barossa Valley [i.e., vineyards 1, 2, 3, and 4 (Northern Grounds), 5, 6, 7, and 8 (Central Grounds), and 13, 14, and 15 (Southern Grounds)] (**Figure 2A**) were selected as Northern and Southern Grounds showed the greatest epigenetic differentiation (**Table 2**). PCoA analysis showed that Central Grounds samples occupied an intermediate Eigen space between Northern and Southern Grounds samples with coordinate 1 (24% of the observed variability) representing the North–South axis (**Figure 2B**). Moreover, Mantel test showed a significant (*P* = 0.0003) positive correlation (*R*^2^ = 0.3066) between pairwise vineyard epigenetic and GeoDs (**Figure 2C**). Then, Mantel test analysis was implemented to compare the observed molecular differences against environmental variables. Differences in vineyard altitude showed a small but significant positive correlations (*R*^2^ = 0.1615, *P* = 0.013) with PhiPT values between vineyards (**Supplementary Figure S3**). We then investigated if clone and vineyard management systems could be contributing to this correlation, by comparing the epigenetic/GeoDs correlation of 10 vineyards planted with clone 1654 [vineyards 1 and 4 (Northern Grounds), 7 (Central Grounds), 9 and 12 (Eastern Ridge), 15 (Southern Grounds) 16, 17, 18, and 19 (Western Ridge) (**Figure 3A**)] and of six vineyards planted with the same clone (1654) and trained using the same pruning system (i.e., spur pruning) [vineyards 1 (Northern Grounds), 7 (Central Grounds), 9 (Eastern Ridge), 15 (Southern Grounds), 16 and 19 (Western Ridge) (**Figure 4A**)]. Again, PCoA shows that the main contributor (23–24%) to the detected variability is associated to the distribution of the vineyards on the N–S axis. Mantel test showed a positive correlation for both epigenetic/GeoD comparisons, however, although both correlations were significant (*P* < 0.05), the correlation among vineyards pruned using the same system (**Figures 4B,C**) was higher than that observed when all pruning systems were incorporated in the analysis (**Figures 3B,C**).

### msGBS Analysis of Genome/Methylome Differentiation between Northern, Central, and Southern Grounds

TASSEL 3 was then implemented on msGBS data for SNP calling from 99 samples collected in 11 vineyards in the Northern, Central, and Southern Grounds sub-regions. This generated a total of 8,139 SNPs of which 4,893 were present in at least 80% of the sequenced samples. PCA analysis using filtered SNPs showed very low level of genetic structure, with only five plants from vineyard 3 (Northern Grounds) separating from the rest (**Supplementary Figure S4A**). However, this clustering was not supported by DAPC (i.e., the optimal clustering solution should correspond to the lowest BIC) which indicated the optimal number of clusters for this data set is 1 (**Supplementary Figure S4B**) suggesting a lack of genetic structure in the vineyards/regions analyzed.

**FIGURE 5:**
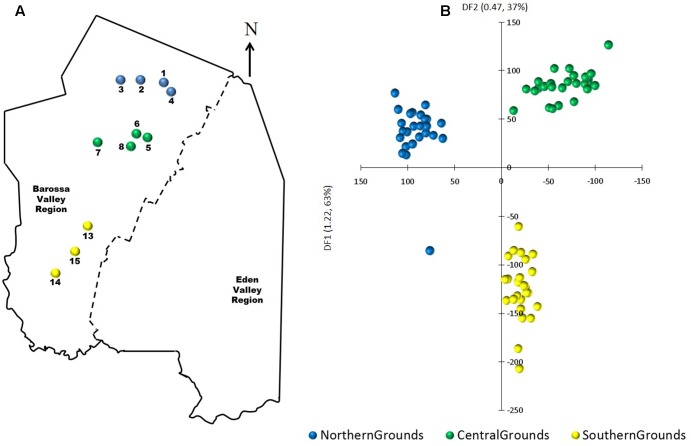
Analysis of the correlation between epigenetic differentiation and geographic location of vineyards planted along the Barossa Valley North–South axis. **(A)** Location of the Barossa Valley vineyards from the three sub-regions distributed along the Barossa Valley North–South axis; Northern Grounds (blue), Central Grounds (green), and Southern Grounds (yellow). Arrow indicatesthe direction of geographic North. **(B)** Principal Components-Linear Discriminant Analysis (PC-LDA) representing epigenetic differences between leaf samples collected from nine plants/vineyard. Percentage of the variability capture by each differential factor (DF) is shown in parenthesis. PC-LDA were based on read number of loci obtained from msGBS profiles.

Principal components-linear discriminant analysis (PC-LDA) was then used to visualize differences in DNA methylation detected using msGBS. DNA methylation profiles clustered samples by their sub-region of origin, with Northern and Central Grounds being separated by differential factor (DF1) from Southern Grounds while DF2 separated Northern from Central Grounds (**Figure 5**). These results were supported by the higher number of DMMs found when comparing samples from Southern to samples from Central or Northern Grounds than when comparing Northern to Central (**Table 3**).

**Table 3:** Identification of DMMs, DMGs, and GO terms (DMGOs) between sub-regions in Barossa Shiraz.

	Differentially methylated markers	Differentially methylated genes		Differentially methylated GO terms	
		
		Hypomethylated genes	Hypermethylated Genes	Hypomethylated GO terms	Hypermethylated GO terms
NG vs. CG	7465	374	178	327	204
SG vs. CG	15276	691	2382	520	832
SG vs. NG	12911	522	2094	500	815


*Cells contain the number of DMMs, DMGs, or DMGOs detected in each pairwise comparison. Differential methylation between sub-regions was calculated using msGBS data from nine Shiraz plants per vineyard [Northern Grounds (NG): four vineyards, Central Grounds (CG): four vineyards and Southern Grounds (SG); three vineyards]. Directionality of methylation (i.e., hyper/hypomethylation) indicates an increase or decrease in DNA methylation in the second region compared to the first region in each pairwise comparison.*

**FIGURE 6:**
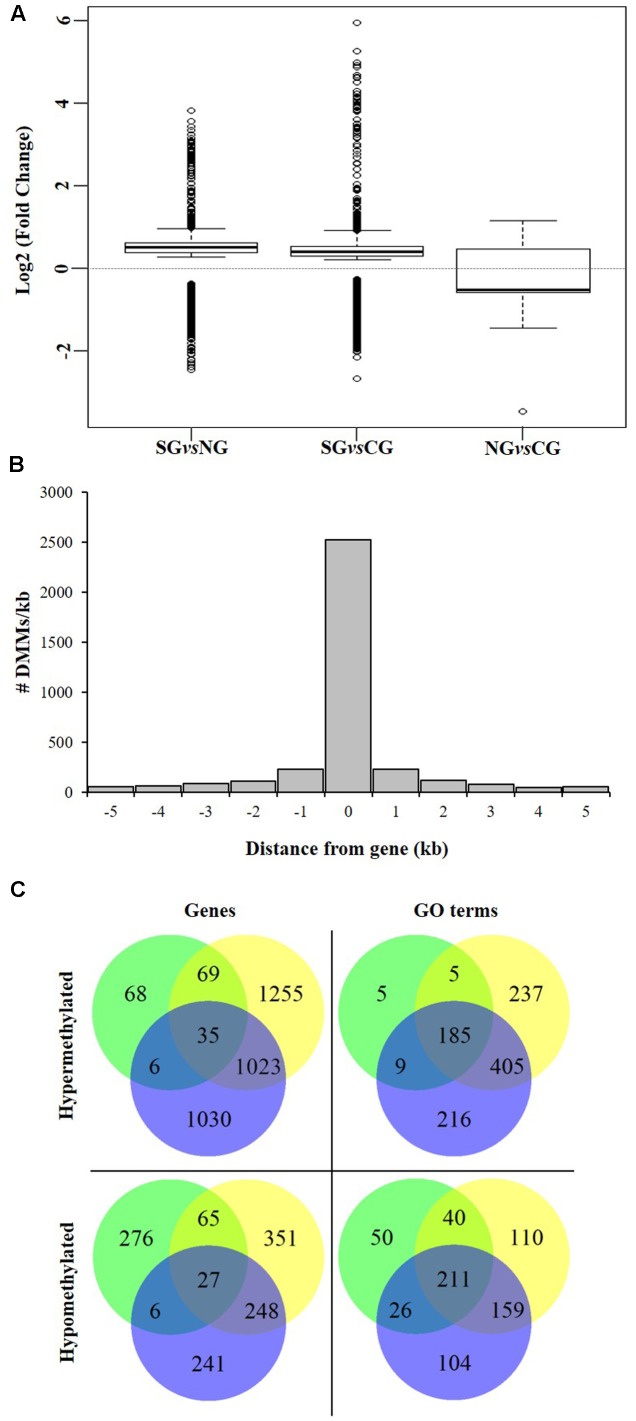
Analysis of DMGs and GO terms (DMGOs) among three wine sub-regions in Barossa Shiraz. Genes were considered differentially methylated if located within 5 kb of at least one DMM (FDR < 0.01). DMMs were generated using msGBS on nine plants per vineyard (Northern Grounds: four vineyards, Central Grounds: four vineyards, and Southern Grounds; three vineyards). **(A)** Directionality of methylation differences between regions. Boxplots show the distribution of the intensity of changes in DNA methylation level between regions, represented here as the fold-change (2 power *log*2FC) in read counts for a given msGBS markers between two regions. Median shows the direction of the methylation flux at a whole genome level in each region comparison (i.e., positive medians indicate a global increase in DNA methylation (hypermethylation) while negative medians indicate a global decrease in DNA methylation (hypomethylation) in the second region in the comparison (e.g., Northern Grounds is hypermethylated compared to Southern Grounds). **(B)** Distribution of 3598 region specific DMMs around genes. Columns –5 to –1 and 1 to 5 represents the number of DMMs per kb around *V. vinifera* genes. Column 0 indicates the number of DMMs within the coding sequence (i.e., between the transcription start and end sites) of *V. vinifera* genes. **(C)** Shared DMGs and DMGOs between regional comparisons. Venn diagrams show the number of unique and shared DMGs and DMGOs between each regional pairwise comparison (i.e., Blue: hyper/hypomethylated genes and GOs in Northern Grounds compared to Southern Grounds; Yellow: in Central Grounds compared to Southern Grounds; and Green: in Central Grounds compared to Northern Grounds).

We next investigated the association of the detected DMMs to annotated protein-coding genes in the grapevine genome by surveying their location and density within and flanking such genomic features. A total of 3,598 genes were deemed differentially methylated (i.e., presented one or more DMMs within 5 kb of the TSS or the TSE) or within genes (**Table 3**). Quantification of such DNA methylation changes showed that, in average, methylation levels are higher in the northern most region in each comparison (i.e., NG > CG > SG) (**Figure 6A**). The majority of detected DMMs associated to a gene were present in the body of the gene and the number of DMMs decreased symmetrically with distance from the TSS and the TES (**Figure 6B** and Supplementary Tables S4–S6). Finally, as observed with all DMMs, the comparison between Northern and Central Grounds samples showed the lowest number of DMGs (**Table 3**, **Figure 6C**, and Supplementary Tables S4–S6).

**FIGURE 7:**
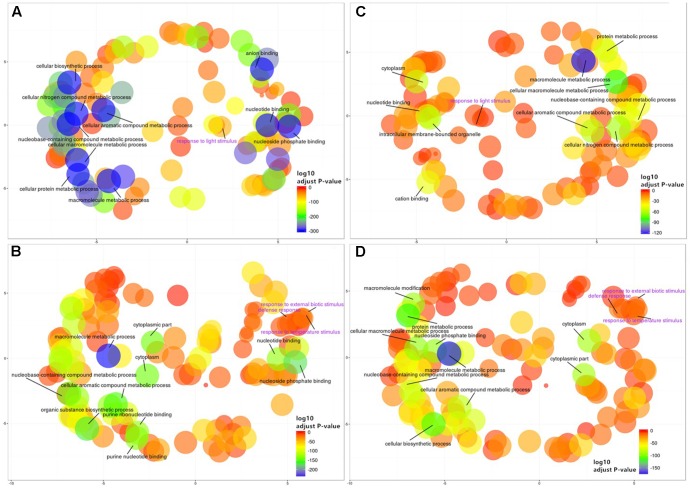
REViGO semantic analysis of differentially methylated GO terms shared by all three regional pairwise comparisons. Functional enrichment of GO-terms was carried out for the genes deemed differentially methylated (DMGs) hypermethylated (185) **(A,C)** or hypomethylated (211) **(B,D)** in Northern Grounds compared to Southern Grounds **(A,B)** and Central Grounds compared to Northern Grounds **(C,D)** using GO.*db* and *annotate* and summarized using REViGO. Bubble color indicates the *p*-value for the FDRs (the first 10 terms are labeled with legends in black. A detailed list of all GO terms containing DMGs has been supplied as a Supplementary Tables S7 and S8); circle size indicates the frequency of the GO term in the underlying GO database (bubbles of more general terms are larger).

To gain further insight into the functional implications of the DNA methylation differences detected between sub-regions, we used *GO.db* (Carlson, 2016) and *annotate* (Gentleman, 2016) to assign 1,144 unique GO terms to the observed DMGs (adjusted *P*-value < 0.05). As observed with DMMs and DMGs the comparison between Northern and Central Grounds samples showed the lowest number of GO terms containing DMGOs (**Table 3**, **Figure 6C**, and Supplementary Tables S4–S6). REViGO semantic analysis of GO terms shared by all three pairwise regional comparisons (**Figure 7**) showed an increase of gene enrichment (i.e., a decrease in adjusted *P*-values) with GeoD (e.g., see **Figure 7** for comparisons between Northern Grounds and Southern Grounds (A,B) and Central Grounds and Northern Grounds (C,D). Three hundred and eleven DMGs (8.6% of the total) were allocated in GO terms associated to response to environmental stimulus (161 and 150 abiotic and biotic challenges, respectively) (**Figure 7** and Supplementary Tables S7, S8), which included GO terms in the semantic space of plant response to light, temperature, osmotic/salt stress and defense to biotic stimulus.

## Discussion

In this study, we analyzed the effect of growing region on the methylation profiles of Shiraz plants using MSAP and msGBS. Both techniques use methylation sensitive enzymes to discover DNA methylation polymorphisms between samples. Although the use of restriction enzymes has the obvious limitation of being capable of detecting such polymorphisms only on the context of their recognition sequence, the technology has been extensively validated over the last 20 years and is considered highly reliable (Yaish et al., 2014; Li et al., 2015; Guevara et al., 2017).

### Grapevine DNA Methylation Patterns Are Region Specific

Analysis of *Hpa*II and *Msp*I generated MSAP profiles showed that the methylation profiles of the six different sub-regions were significantly different (*P* < 0.05) in 25 of the 30 possible pairwise comparisons (**Table 2**). Variability among vineyards and sub-regions was higher in *Msp*I generated profiles (17 and 2%) than in *Hpa*II profiles (8 and 1%), indicating that the detected regional epigenetic differences are, at least partially, sequence context specific (Tricker et al., 2012; Meyer, 2015). Calculated PhiPT values showed low levels of molecular differentiation between sub-regions, even when those differences were statistically significant (**Table 2**). This could be explained by the high proportion of the total variability associated to differences between individual plants (81–91%) compared to 1–2% associated to differences between sub-regions. Such high levels of molecular differentiation between individuals could be due to the random accumulation of somatic variation with age, which can be genetic or epigenetic in nature. A specific limitation of MSAPs is its inability to distinguish between a fully methylated site from a site containing a genetic mutation (Yaish et al., 2014). PCA of genetic polymorphisms detected using msGBS results showed a high level of genetic variability between plants (**Supplementary Figure S4A**) which is characteristic of long living plants in general (Baali-Cherif and Besnard, 2005) and in grapevine in particular (Torregrosa et al., 2011). However, DAPC did not detect any sample clustering associated to their sub-region of origin (**Supplementary Figure S4B**) indicating that genetic diversity is not structured in a geographic manner. Although both genetic and epigenetic somatic variation can be random (Vogt, 2015), different growing conditions will differentially affect the DNA methylation profiles of otherwise genetically identical individuals (Consuegra and Rodríguez López, 2016) as previously shown on clonally propagated *Populus alba* (Guarino et al., 2015). It is, therefore, not surprising to find that epigenetic profiling was a better predictor of sample origin than genetic profiling alone both using MSAP data (**Table 2** and **Supplementary Figure S1**) or msGBS data (**Figures 5**–**7** and **Supplementary Figure S4**). This suggests that although genetic differences between regions or vineyards can partly contribute to the observed molecular differentiation between vineyards/sub-regions, epigenetic differences are the major driver of such differentiation.

Previous studies have shown that in some instances clonal variability in grapevine is better explain by epigenetic than genetic differences (Imazio et al., 2002; Schellenbaum et al., 2008; Ocaña et al., 2013). It is therefore possible, that the epigenetic differences observed here are not associated to regional environmental differences but that they were present since the time of planting or due to environmental variations that may have occurred at the time of the sampling. For this reason, further research including information from more than one season is needed to validate the DNA methylation differences between regions observed here. However, the fact that all regions studied here contained three to four vineyards planted at different times and with different clones makes plausible to infer that the region specific epigenetic markers detected are not a reflection of the epigenetic profiles of the plants at the time of planting. Moreover, the positive correlation between molecular distance and GeoD observed on the vineyard pairwise comparisons provides further support for the influence of different environments on the epigenetic profiles of the plants in this study.

Samples collected from vineyards in the Southern Grounds presented the highest levels of both genetic and epigenetic diversity (**Table 1**). These vineyards also presented higher levels of differentiation when inter-vineyard variability was analyzed (**Supplementary Figures S2G,H**), suggesting a major contributor to the observed molecular variability between vines in the Southern Grounds is linked to the vineyard of origin. Taken collectively, these results suggest that the specific growing conditions from each subregion impose DNA methylation patterns on grapevine plants specific for each region as previously shown both in cultivated (Guarino et al., 2015) and wild plant populations (Fonseca Lira-Medeiros et al., 2010). Not surprisingly, and contrary to what has been shown in natural plant populations (Fonseca Lira-Medeiros et al., 2010; Róis et al., 2013), no clear negative correlation between genetic and epigenetic diversity was observed in the studied vineyards. This is most probably due to the intensive phenotypic selection to which grapevine cultivars have been under since domestication and the relative low levels of environmental disparity to which vines growing in the same vineyard are exposed to.

### Differences in Altitude and Pruning System Correlate with Vineyard Epigenetic Differentiation

Principal coordinate and Mantel test analysis showed that the correlation between epigenetic and GeoD between vineyards on the North–South axis of the Barossa Valley (**Figure 2A**) was significant (*P* = 0.0003) (**Figure 2C**) and that the main contributor to the observed epigenetic differences was the position of the studied vineyards along the N–S axis (**Figure 2B**). This suggests that environmental differences between locations could be contributing to the observed molecular differences between sub-regions or vineyards (**Figure 3**). Moreover, the correlation (*R*^2^ = 0.3066) between epigenetic and GeoD among vineyards planted with clone 1654 on the N–S axis (**Figure 2**) supports the Shannon diversity analysis that indicate that the different genetic backgrounds used in this study do not greatly affect the epigenetic differences observed between regions (**Table 1**). Conversely, differences in vineyard altitude appear to be a contributor to the detected epigenetic differentiation between vineyards (**Supplementary Figure S3**). Previous work has shown that sun exposure can have significant effects both in berry metabolomic profiles (Son et al., 2009; Tarr et al., 2013) and on the epigenetic profiles of plants growing in different environments (Guarino et al., 2015). Although altitude does not necessarily affect sun exposure, it can have a profound effect on the UV levels experienced by plants (approximately 1% increase every 70 m gain in altitude). Our results suggest that, although DNA methylation in and around genes changes in both directions (hyper- and hypo-methylation), on average, it increases with altitude (i.e., NG > CG > SG; vineyard average altitude 301, 277, and 236 m, respectively) (**Figure 6A**).

Due to the nature of the msGBS approach used here, all sequenced DMMs are in the CHG context. Global methylation levels on this context varies widely between plant species (9.3% in *Eutrema salsugineum* to 81.2% in *Beta vulgaris*) (Niederhuth et al., 201*6)*. In *V. vinifera*, genome-wide weighted CHG methylation level is 20.4%, more than double than that found in *A. thaliana* (Niederhuth et al., 2016). Although the analysis of DNA methylation has traditionally focus on the CG context, CHG differential methylation has been reported to be more prominent than CG differential methylation in other perennial crops (i.e., Apple, *Malus domestica*) (Daccord et al., 2017). In this study, the majority of detected DMMs associated to a gene were present in the body of the gene (**Figure 6B**). The function of gene body methylation (GbM) is not yet well understood (Zilberman, 2017) and recent studies have shown that GbM can be lost over evolutionary time with no deleterious consequences, suggesting that it might not be required for plant viability (Bewick et al., 2016). However, plant accessions with higher average GbM have been shown to have higher average expression of gene body methylated genes (Dubin et al., 2015; Wang et al., 2015). Moreover, GbM has also been proposed as a regulator of alternative splicing (Wang et al., 2016) and suppressor of intragenic cryptic promoters and transposable element (Maunakea et al., 2010). In particular CHG GbM has been associated to the silencing of genes lacking CG GbM (Niederhuth and Schmitz, 2017) and to the repression of splicing in maize (Regulski et al., 2013). Remarkably, global methylation levels of the CG, CHG, and CHH contexts has been proposed to be an adaptive trait to environmental variables such as latitude, aridity and photosynthetically active radiation temperature, respectively (Dubin et al., 2015). It is, therefore, tempting to speculate that the differences in GbM observed between regions reflect plant adaptation to their local environments that could be affecting alternative splicing, which has been itself been proposed as an adaptive mechanism (Ast, 2004).

Functional analysis of the DMGs between sub-regions generated GO terms associated to plant response to light stimulus (Supplementary Table S8). More importantly, the number of genes associated to such GO terms was higher in comparison between regions with bigger differences in altitude [74 and 46 genes in comparison SG vs. NG (65 m difference) and SG vs. CG (41 m), respectively] than in the pairwise comparison with lower difference in altitude (6 genes NG vs. CG (24 m)]. Although this positive polynomial grade 2 correlation (*R*^2^ = 1) was generated using only three data points, it is tempting to speculate that differences in light incidence due to differences in altitude are triggering the observed changes in DNA methylation in response to light stimulus genes. Especially when previous work has shown that, in grapevine leaves, increased UV levels trigger the synthesis of non-flavonoid phenolics such as resveratrol (Sbaghi et al., 1995; Teixeira et al., 2013). Interestingly, DNA methylation has been previously linked to the regulation of the gene VaSTS10, which controls the synthesis of resveratrol in *Vitis amurensis* (Kiselev et al., 2013; Tyunin et al., 2013).

The correlation between epigenetic and GeoDs observed between vineyards planted with clone 1654 and pruned with the same method (spur pruning) (**Figure 4**) was reduced when all vineyards planted with clone 1654 were considered irrespectively of the pruning system used (**Figure 3**). This suggests that differences in pruning system, in conjunction with environmental conditions, might be contributing to the epigenetic differences observed between vineyards and sub-regions in this study. However, further research on the effect of the observed change in DNA methylation with vineyard altitude and pruning on gene expression are needed to validate the hypothesis that such changes might be regulating plant adaptation to such environmental cues.

## Conclusion

Vintage, geographic location, and vineyard management have been shown to influence both vegetative growth (Jackson and Lombard, 1993) and fruit composition in grapevine (Roullier-Gall et al., 2014). In light of the results shown here, we propose that epigenetic processes in general and DNA methylation in particular, could constitute an important set of molecular mechanisms implicated in the effect that provenance and vintage has, not only on plant vegetative growth, but also on fruit and wine quality. It is important to stress that since global patterns of DNA methylation are tissue/organ specific (Rodríguez López et al., 2010), the observed differences in DNA methylation profiles between plants growing in different regions can only be taken as indicative of those occurring in leaves. However, the effect of the environment on the epigenetic profiles of different tissues in plants reflects their mode of development. That is, unlike mammals, plants growth and organ formation occurs from stem cell populations in the meristems (Pikaard and Mittelsten Scheid, 2014). Previous studies (e.g., Verhoeven et al., 2010; Tricker et al., 2013a) have shown that environmentally induced markers detected in leaf tissue can be found on subsequent generations. This indicates that the DNA methylation markers observed in leaves were also present in the meristematic tissue that ultimately produced the reproductive organs. For this reason, it is plausible to expect that region-specific markers detected in grapevine leaves, could also be present in other organs such as berries since these are originated from the same shoot apical meristems as leaves. Although preliminary, our results open the door to speculate that epigenetic priming (Tricker et al., 2013b) could act as a form of epigenetic memory of the vineyard’s environment that would ultimately contribute, at least partially, to the uniqueness of wines produced in different regions. Testing this hypothesis will require the integrative analysis of fruit DNA methylation, gene expression, and metabolite composition data from multiple seasons to account for the effect of inter-annual climatic variations on fruit composition (Fabres et al., 2017).

## Author Contributions

HX and MK carried out the experiments and contributed to data analysis. NS performed gene ontology analysis on msGBS data. KT performed TASSEL analysis on msGBS data. TC, MG, JB, AM, and JS contributed to the design of the research project. RDB and CC contributed to the design of the research project, site selection and collection of material. CL contributed to the design of the research project, data analysis and drafted the manuscript. All authors read and contributed to the final manuscript.

## Conflict of Interest Statement

The authors declare that the research was conducted in the absence of any commercial or financial relationships that could be construed as a potential conflict of interest.

## Footnotes

1 http://adegenet.r-forge.r-project.org/

2 https://github.com/BenjaminAdelaide/msgbsR

3 http://plants.ensembl.org/Vitis_vinifera/

4 http://plants.ensembl.org/biomart/martview/

## Supplementary Material

**FIGURE S1 |** Analysis of genetic and epigenetic differences within Barossa Valley sub-regions. PCoAs represent variability of NML (genetic variability) **(A,C,E,G,I,K)** and of MSL (epigenetic variability) **(B,D,F,H,J,L)** as classified by the msap (v. 1.1.8) software (Pérez-Figueroa, 2013) of leaf samples in vineyards from Northern Grounds **(A,B)**, and the Barossa Valley’s Western Ridge Central Grounds **(C,D)**, Eastern Edge **(E,F)**, and Southern Grounds **(G,H)**, Western Ridge **(I,J)**, and Eden valley **(K,L)**. Coordinates 1 and 2 are shown with the percentage of variance explained by them. Points represent individuals from each vineyard. Vineyard code (NG, CG, EE, SG, WR, and EV) is shown as the centroid for each vineyard. Ellipses represent the average dispersion of those poinst around their center. The long axis of the ellipse shows the direction of maximum dispersion and the short axis, the direction of minimum dispersion.

**FIGURE S2 |** Analysis of regional genetic and epigenetic diversity. Red symbols indicate samples analyzed using genetic information only, black symbols represent samples analyzed using epigenetic information only according to the R package for statistical analysis of MSAP data “msap.” PCoAs were calculated using MSAP profiles generated from gDNA extracted from E-L 7-stage leaves (Coombe, 1995) of 88 grapevine plants grown in vineyards located in the six Barossa Valley wine sub-regions (**A**, Northern Grounds; **B**, Central Grounds; **C**, Southern Grounds; **D**, Eastern Edge; **E**, Western Ridge; **F**, Eden valley) (four individual plants per vineyard) using restriction enzyme combinations *Msp*I/*EcoR*I and *Hpa*II/*EcoR*I.

**FIGURE S3 |** Analysis of the correlation between epigenetic differentiation and environmental differences among vineyards planted along the Barossa Valley North–South axis: Mantel test analysis of the correlation between pairwise epigenetic distance (MspI PhiPT) and differences in altitude between vineyards. Shown equations are the correlation’s *R*^2^ and the Mantel test significance (*P*-value was estimated over 9,999 random permutations tests). PhiPT values were based on presence/absence of 215 loci obtained from MSAP profiles generated using *Msp*I.

**FIGURE S4 |** Analysis of the grapevine genetic diversity in vineyards planted along the Barossa Valley North–South axis. **(A)** PCA representing genetic structure calculated using 4,893 high quality SNPs (i.e., present in at least 80% of the samples) in genomic DNA collected from 11 vineyards [Northern Grounds (blue) four vineyards, Central Grounds (green) four vineyards, and Southern Grounds (yellow) three vineyards (nine plants/vineyard)]. **(B)** Identification of the optimal number of genetic clusters present within the three sub-regions compared using BIC as implemented by DAPC using adegenet 2.0.0 (i.e., the optimal clustering solution should correspond to the lowest BIC).

## Abbreviations

AMOVA: analysis of molecular variance
BAM file: Binary Alignment/Map file
BIC: Bayesian Information Criterion
CPM: counts per million
DAPC: discriminant analysis of principal components
DMG: differentially methylated gene
DMM: differentially methylated marker
FDR: false discovery rate
gDNA: genomic DNA
GeoD: geographic distance
GO: gene ontology
kb: kilobase
log_2_FC: logarithm 2 of fold change
MSAPs: methylation sensitive amplified polymorphisms
msGBS: methylation sensitive genotyping by sequencing
MSL: methylation sensitive polymorphic loci
NML: non-methylated polymorphic loci
PCA: principal component analysis
PCoA: principal coordinate analysis
PhiPT: measurement of epi/genetic diversity among populations
SI: Shannon’s diversity index
SNP: single nucleotide polymorphism
TES: transcription end site
TSS: transcription start site
