# Supplementary figures and images for: Global DNA Methylation Patterns Can Play a Role in Defining Terroir in Grapevine (Vitis vinifera cv. Shiraz)
Source: Front Plant Sci. 2017 Oct 30;8:1860. doi: 10.3389/fpls.2017.01860 (PMC5670326; doi:10.3389/fpls.2017.01860)

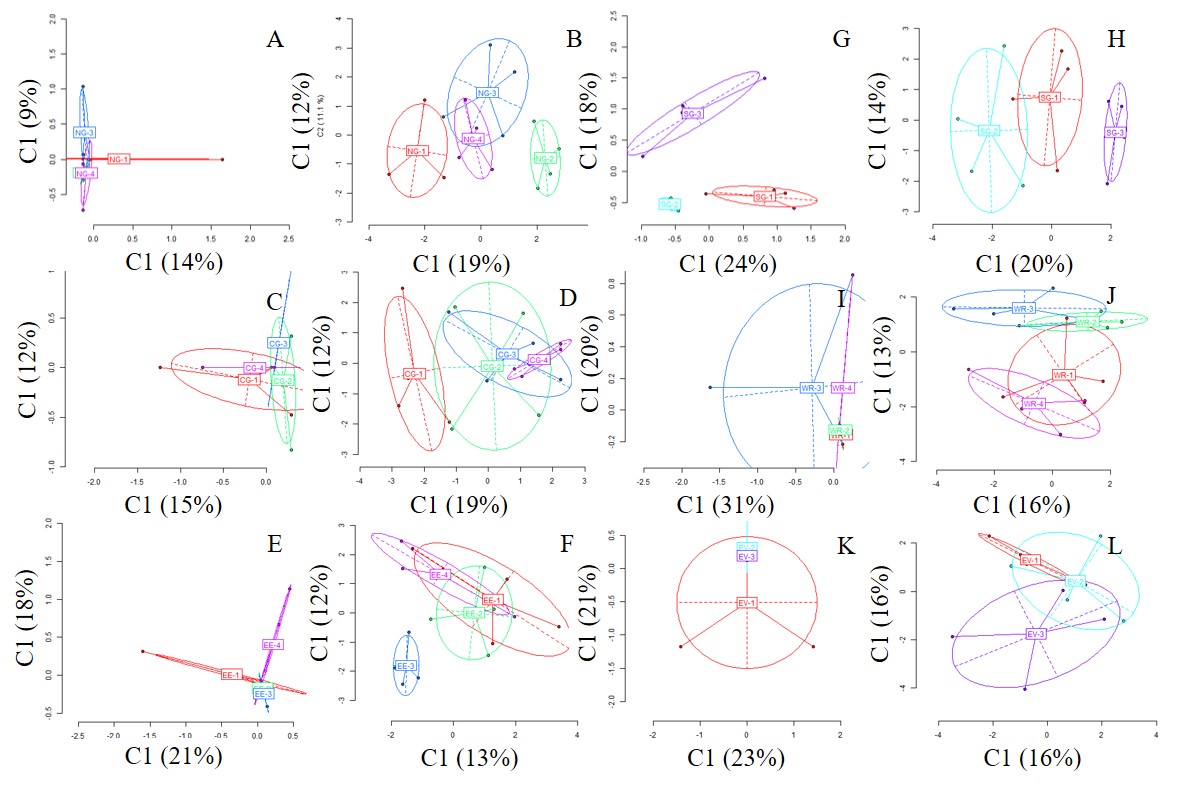

Supplement: Supplementary file 1 [file Image_1.JPEG]

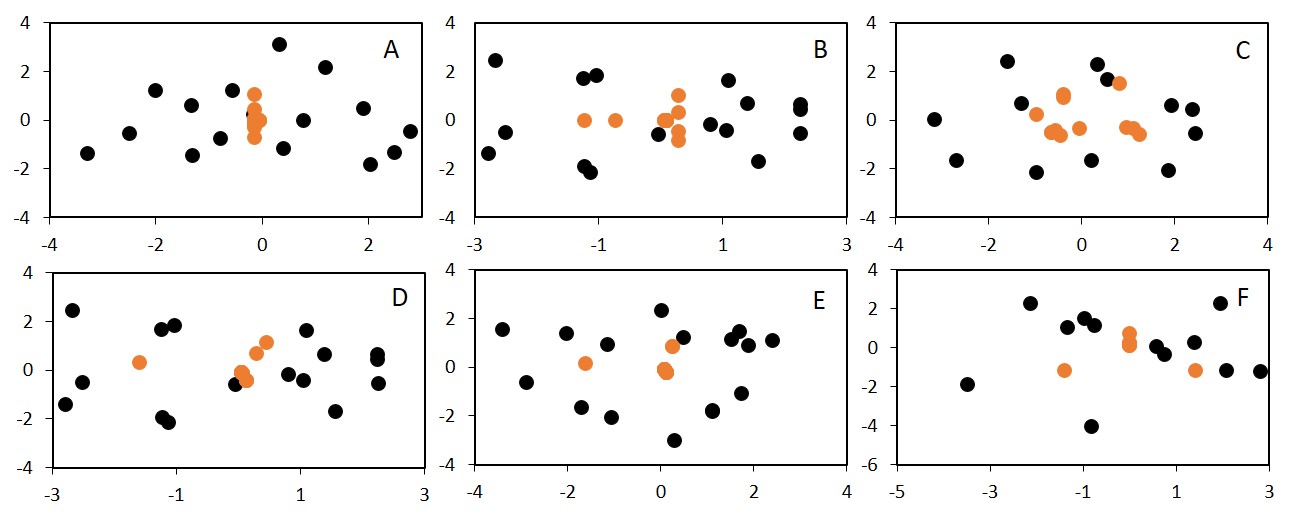

Supplement: Supplementary file 2 [file Image_2.JPEG]

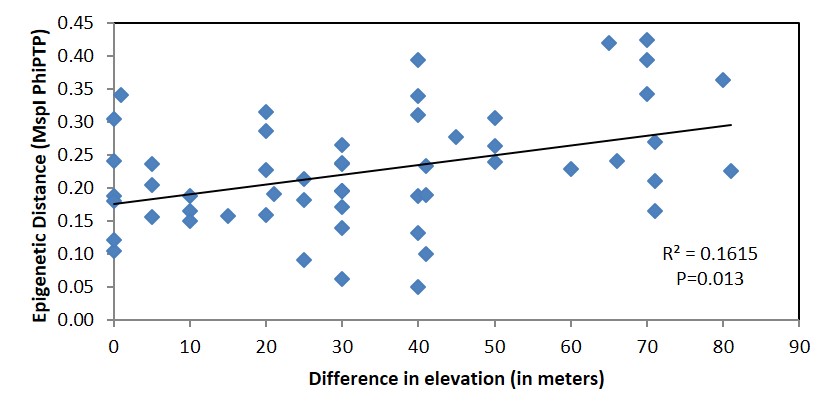

Supplement: Supplementary file 3 [file Image_3.JPEG]

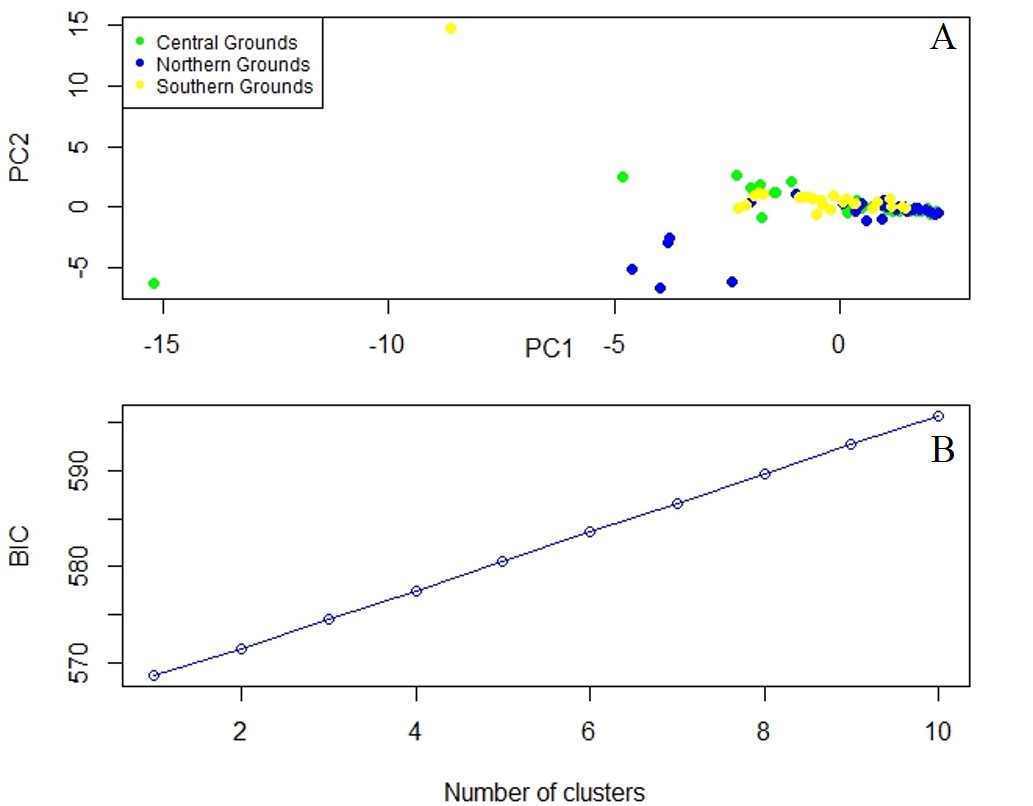

Supplement: Supplementary file 4 [file Image_4.JPEG]
