# Supplementary material for: Global DNA Methylation Patterns Can Play a Role in Defining Terroir in Grapevine (Vitis vinifera cv. Shiraz)
Source: Front Plant Sci. 2017 Oct 30;8:1860. doi: 10.3389/fpls.2017.01860 (PMC5670326; doi:10.3389/fpls.2017.01860)
Supplement: Supplementary file 6 [file Table_2.docx]

**Table S2:** Sequences of oligonucleotide used for MSAP. Selective bases in the primers used during the preselective and selective amplifications are highlighted in bold. Unique msBGS barcode bases are represented as X.

| **Oligo name** | **Function** | **Sequence** |
| --- | --- | --- |
| *Hpa*II/*Msp*I adaptor | Reverse Adaptor | CGCTCAGGACTCAT |
| *Hpa*II/*Msp*I adaptor | Forward Adaptor | GACGATGAGTCCTGAG |
| *Eco*RI adaptor | Reverse Adaptor | AATTGGTACGCAGTCTAC |
| *Eco*RI adaptor | Forward Adaptor | CTCGTAGACTGCGTACC |
| Pre- EcoRI | Preselective primer | GACTGCGTACCAATTC**A** |
| Pre- *Hpa*II/*Msp*I | Preselective primer | GATGAGTCCTGAGCGG**C** |
| *Eco*RI Selective Primer | Selective primer | GACTGCGTACCAATTC**ACG** |
| *Hpa*II/*Msp*I Selective Primer | Selective primer | GATGAGTCCTGAGCGG**CAA** |
| *Msp*I msGBS barcoded adaptor | Reverse Adaptor | CGXXXXAGATCGGAAGAGCGTCGTGTAGGGAAAGAGTGT |
| *Msp*I msGBS barcoded adaptor | Forward Adaptor | ACACTCTTTCCCTACACGACGCTCTTCCGATCTXXXXX |
| *Eco*RI msGBS Y adaptor | Reverse Adaptor | CGAGATCGGAAGAGCGGTTCAGCAGGAATGCCGAG |
| *Eco*RI msGBS Y adaptor | Forward Adaptor | CTCGGCATTCCTGCTGAACCGCTCTTCCGATCT |
| *Msp*I msGBS primer | Sequencing library primers | AATGATACGGCGACCACCGAGATCTACACTCTTTCCCTACACGACGCTCTTCCGATCT |
| *Eco*RI msGBS primer |  | CAAGCAGAAGACGGCATACGAGATCGGTCTCGGCATTCCTGCTGAACCGCTCTTCCGATCT |
