# Supplementary material for: Global DNA Methylation Patterns Can Play a Role in Defining Terroir in Grapevine (Vitis vinifera cv. Shiraz)
Source: Front Plant Sci. 2017 Oct 30;8:1860. doi: 10.3389/fpls.2017.01860 (PMC5670326; doi:10.3389/fpls.2017.01860)
Supplement: Supplementary file 7 [file Table_3.docx]

**Table S3:** Ratio of methylation status types on the CCGG context detected analysed using *msap* R package for each of the studied wine subregions.

| Methylation status of loci | Wine subregion | | | | | |
| --- | --- | --- | --- | --- | --- | --- |
|  | Northern Grounds | Central Grounds | Southern Grounds | Eastern Edge | Western Ridge | Eden Valley |
| Internal cytosine methylation | 0.167 | 0.1677 | 0.185 | 0.1843 | 0.1854 | 0.1827 |
| Unmethylated | 0.1547 | 0.1653 | 0.218 | 0.1889 | 0.2115 | 0.2147 |
| Hemimethylated | 0.2105 | 0.2105 | 0.1817 | 0.2161 | 0.202 | 0.1916 |
| Full methyl/SNP | 0.4679 | 0.4566 | 0.4153 | 0.4107 | 0.4011 | 0.411 |
